# Supplementary material for: SARS-CoV-2 RNAemia and Disease Severity in COVID-19 Patients
Source: Viruses. 2023 Jul 16;15(7):1560. doi: 10.3390/v15071560 (PMC10386401; doi:10.3390/v15071560)
Supplement: Supplementary file 1 [file viruses-15-01560-s001.zip › viruses-2498155-supplementary.pdf]

Figure S1

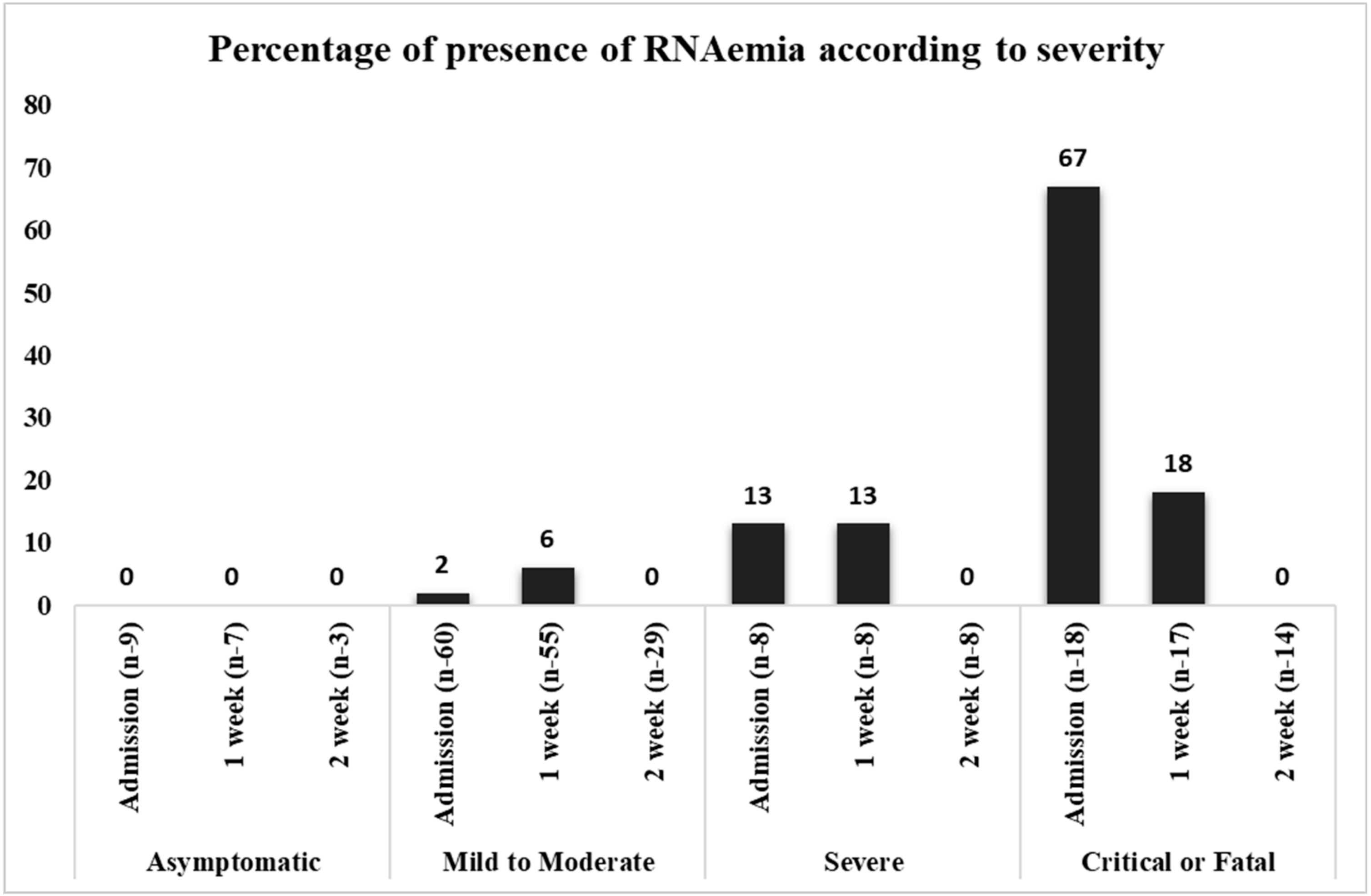

Figure S1. Direct proportion of viral RNAemia with disease severity.
The plasma samples were classified according to Sixth Revised Trial Version of the Novel Coronavirus Pneumonia Diagnosis and Treatment Guidance, and the data are expressed as N (%).

Figure S2

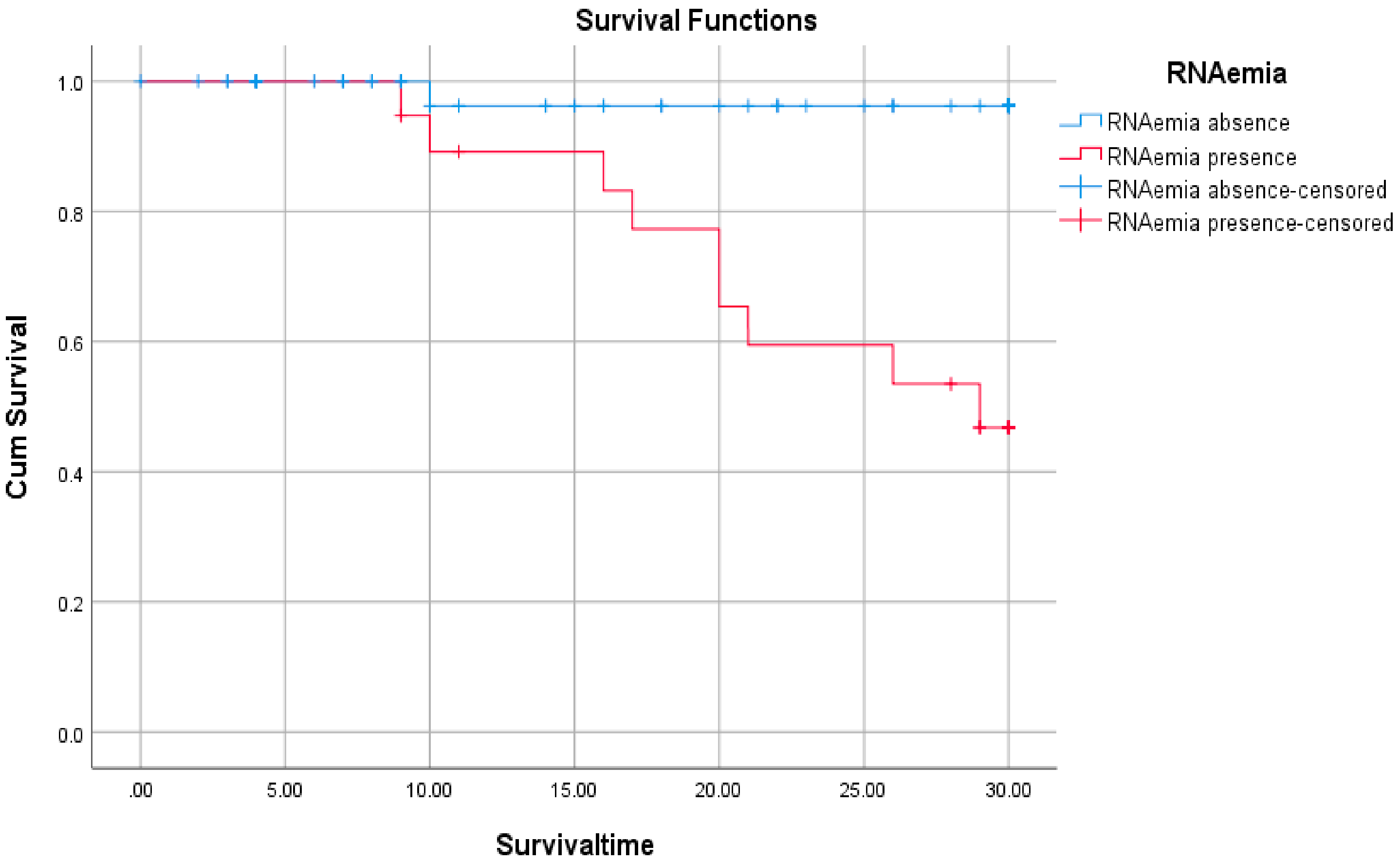

RNAemia absence		RNAemia presence		P Value (Log Rank)
n	30-day Survival Time (Mean, 95% CI)	n	30-day Survival Time (Mean, 95% CI)	
76	29.25(28.22-30.27)	19	24.15(20.82-27.47)	<0.001

Figure S2. Mortality was plotted using patients with RNAemia and non-RNAemia using Kaplan-Meier curve

P-values comparing patients with COVID-19 with evidence of RNAemia to patients without RNAemia were calculated using the Mann-Whitney U test or Fisher's exact test, as appropriate. $P < 0.05$ was considered significant.
